# Random Laser Action in Nd:YAG Crystal Powder

**DOI:** 10.3390/ma9050369

**Published:** 2016-05-13

**Authors:** Jon Azkargorta, Iñaki Iparraguirre, Macarena Barredo-Zuriarrain, Sara García-Revilla, Rolindes Balda, Joaquín Fernández

**Affiliations:** 1 Departamento de Física Aplicada I, Escuela Superior de Ingeniería, Universidad del País Vasco UPV/EHU, Alameda Urquijo s/n, Bilbao 48013, Spain; i.iparraguirre@ehu.eus (I.I.); macarena.barredo@ehu.eus (M.B.-Z.); sara.garcia@ehu.eus (S.G.-R.); rolindes.balda@ehu.eus (R.B.); joaquin.fernandez@ehu.eus (J.F.); 2 Materials Physics Center, CSIC-UPV/EHU, San Sebastián 20018, Spain

**Keywords:** random lasers, crystal powder, neodymium

## Abstract

This work explores the room temperature random stimulated emission at 1.064 μm of a Nd:YAG crystal powder (Neodymium-doped yttrium aluminum garnet) in a very simple pump configuration with no assistance from an internal mirror. The laser threshold energy as a function of pump beam area and pump wavelength has been measured, as well as the temporal dynamics of emission pulses. The absolute energy of stimulated emission and the absolute laser slope efficiency have been measured by using a method proposed by the authors. The results show a surprising high efficiency that takes the low Nd^3+^ ion concentration of the crystal powder into account.

## 1. Introduction

The study of highly scattering materials as laser sources has been a very interesting field over the last 15 or 20 years [1,2,3]. A wide variety of material types have been tested as random emitting laser sources, such as colloidal dye solutions, powdered crystals, polymers, biological samples including human tissues, and many possible applications have been proposed, such as speckle-free laser phosphors, displays, optical or chemical sensors for medical diagnostics, nanoscale lithography, *etc.* [4,5,6,7,8,9]. Many efforts have been made to develop a general theoretical treatment for random lasers (RLs) including spectral behavior [10,11,12], temporal dynamics [13,14,15,16], thresholds [17,18,19,20,21], output energies, *etc.*; however, due to the very wide range of material types, of scattering regimes, of pumped time scales, *etc.*, many fundamental questions remain, and basic features of RLs are still unfixed, such as the absolute emitted energy, the general temporal dynamics, the mode structure, [22,23,24,25] and their dependence with the pump beam area. Therefore, we agree with a statement found in [10] where the authors assert that “a comparison between different experimental studies is very difficult, as the experiments have many parameters not all of which are described completely in literature”.

While classical lasers always use an optical resonator with mirrors to provide feedback to an amplifying medium, RL sources are cavity-less devices using multiple scattering as light feedback; therefore, the emission does not occur in a definite direction, but it is omnidirectional [1,2,3]. However, the emission spectrum is much narrower, the duration of the emission pulse is much shorter, and the intensity of the emission pulse is much higher than the spontaneous emission (by several orders of magnitude). This behavior is similar to the one found in conventional lasers, but instead of being a mere loss factor, the multiple scattering plays a constructive role in RLs, the properties of which must be determined by the competition between diffusion processes, absorption, and amplification.

Nd:YAG is one of the most extensively studied crystals for stimulated emission. Neodymium ion is dispersed as a dopant in the YAG (Y_3_Al_5_O_12_) crystalline matrix in a few percent. Neodymium presents a typical four-level scheme with very efficient absorption bands in the near infrared (NIR) from the ^4^I_9/2_ ground-state level to the ^4^F_J_, excited levels, and very high emission efficiency, especially for the laser ^4^F_3/2_ → ^4^I_11/2_ transition. Furthermore, YAG garnet crystal has good thermal conductivity, very good transparency, hardness, chemical stability, low threshold, and higher efficiency than other host matrices for RE^3+^ ions. These characteristics make it very suitable, for example, for continuous wave (CW) or high-power applications, and one of the most valuable solid-state materials for commercial laser oscillators and amplifiers [26].

However, only a few works have reported Nd:YAG as a RL source. In 1999, Lichmanov *et al.* pumped Nd:YAG crystal powders with an electron beam via scintillator (catodoluminophore) cooled at 77 K, but the parameters of powders were not specified [27]. Feng *et al.* in 2004 obtained laser emission with a “one-mirror structure” and diode pumping, using nanopowders with a 250-nm grain diameter [28]. They proposed a one mirror setup to reduce the lasing threshold because, “without the mirror, no evidence of lasing could be observed” [29]. They described the energy threshold behavior, the temporal behavior in time scales of the order of 200 μs, the emission spectrum, the emitted energy (not in absolute units), thermal effects [30], and a hybrid microchip combining a Nd:YAG transparent ceramic and a Nd:YAG powder tablet in 2005 [31].

In this work, the authors have obtained a 1.064-μm RL emission at room temperature by pumping around 800 nm (^4^I_9/2_ → ^4^F_5/2_ transition) onto the free surface of a 1 mol % Nd-doped YAG crystal powder sample by using a simple experimental setup with no additional mirrors [32,33]. We measured some of the basic features of RL emission: the absolute stimulated emission energy *versus* pump energy and the pump threshold energy as a function of pump spot size and pump wavelength. In spite of the low dopant concentration, an absolute slope efficiency of about 20% has been achieved. The slope efficiency and the pump threshold energy per unit area are constant in a wide range of pumped areas.

## 2. Sample Preparation and Characterization

**Figure 1:**
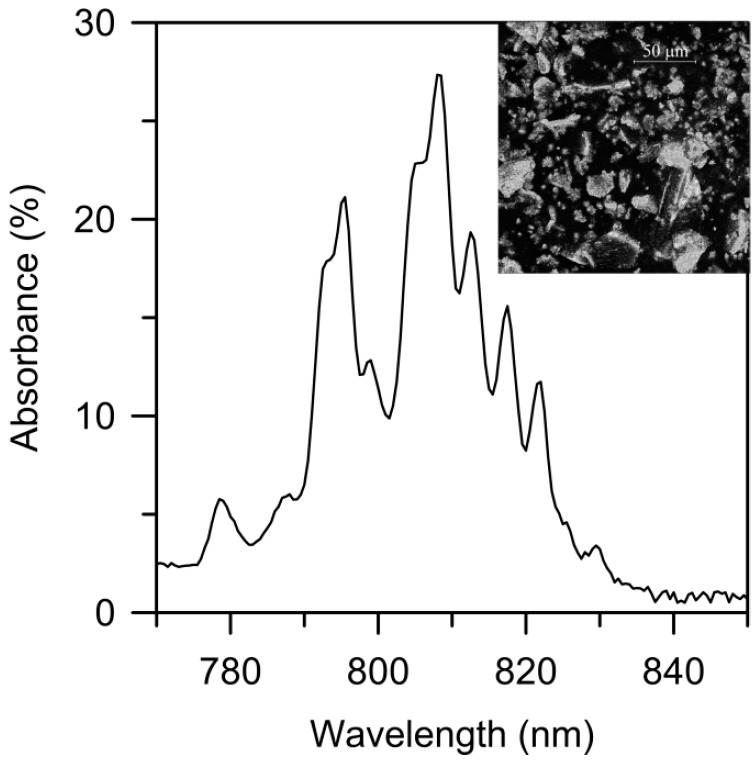
A microscope photograph (inset) and absorbance spectrum of Nd:YAG crystal powder corresponding to the ^4^I_9/2_ → ^4^F_5/2_ transition. The average grain size is of the order of tens of microns.

The Nd-doped YAG crystal powder was obtained by grinding a piece of a commercial Nd:YAG crystal rod, 1% Nd (by Laser Crystal Corp., Lake Hopatcong, NJ, USA) in a Retsch MM200 mechanical mill (Haan, Germany) to an average grain size of the order of tens of microns (inset of Figure 1). In order to estimate the absorbance of the powder sample corresponding to the ^4^I_9/2_ → ^4^F_5/2_ transition, we measured the diffuse reflectance spectrum of the powder by using an integrating sphere coupled to a spectrophotometer (Cary 5 Varian, 0.5 nm spectral resolution, Santa Clara, CA, USA). Figure 1 shows that a maximal absorbance of about 27% occurs around 808.5 nm. It is worth noticing the high absorbance of the sample, the low Nd^3+^ concentration of the crystal powder taken into account. This wavelength was selected as pump wavelength in RL experiments, because it gives the minimal input threshold energy and the maximal laser slope efficiency [32,33].

## 3. Experimental Setup

**Figure 2:**
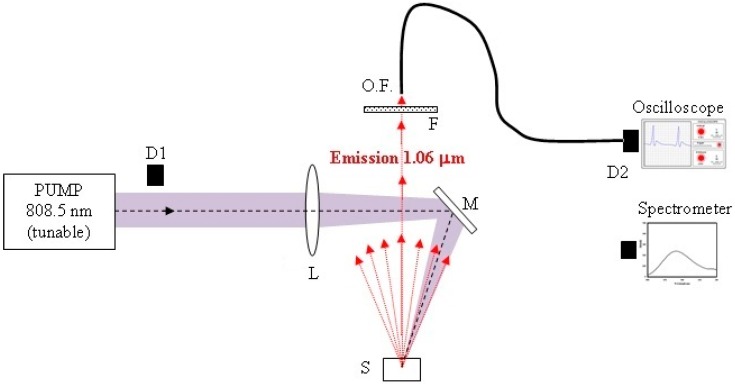
Experimental setup of RL experiments. Pump: tunable Ti:sapphire laser; D1: pump pulse measuring detector; L: movable focusing lens; M: plane mirror; S: sample; F: pump removing filter; O.F.: optical fiber; D2: emission pulse measuring detector, coupled to digital oscilloscope. The fiber end is carried to the spectrometer for spectral measurements.

Figure 2 shows the RL experimental setup. The pump source is a tunable pulsed Ti:sapphire laser (BMI TSA-802, Hersbruck, Germany, 10 ns pulse duration) whose pulses are received by diffusion by a silicon detector D1 (Newport 818-BB-21, Irvin, CA, USA) and driven to a digital oscilloscope (Tektronix TDS7104, Benverton, OR, USA, 10 Gsamples/s). A focusing lens (L) and a plane mirror (M) address the pump beam onto the surface of the powder sample. The L lens (40-cm focal length) can be moved back and forth to adjust the pump beam size (the focal point is farther than the expected focal length of the lens, due to the divergence of the Ti:sapphire laser output beam). The emission is collected in the vertical direction by an optical fiber head (0.5-mm diameter), which drives the stimulated emission to a fast Photodetector, D2 (Newport SIR 5-FC, Irvin, CA, USA), coupled to the oscilloscope. For spectral measurements, the collected emission is driven to a spectrometer (Jobin Ybon TRIAX 190, Edison, NJ, USA). The temporal dispersion introduced by the fiber is negligible within our resolution, given the real numerical aperture used in the measurement system, taking into account the pump diameter (about 1 mm) and sample-fiber distance, 0.2 m.

In order to calibrate the pump pulse energy, a laser energy meter (Ophir PE25BF-V2 ROHS, Irvin, CA, USA) was located at the position of the sample (defocusing the L lens enough to avoid damaging the detector), and its output was compared to the measurement provided by the D1 detector. The pump spot size must be accurately measured to calculate pump energy densities. For that purpose, a 5-cm focal length lens was located in the vertical direction parallel to the emitting surface, and a Laser Beam Profiler (Newport LBP-3, Irvin, CA, USA) was positioned at the image plane. The output emission was also calibrated locating a low-energy laser meter (Ophir PE10-SH-V2, Irvin, CA, USA) in the vertical direction above the emitting powder, close to the optic fiber head. Assuming a Lambertian emission, or cosine law, it is possible to estimate the total output energy in absolute units with the relation [32,33]:

(1) $$E_{out}={\left(\frac{r}{R}\right)}^{2}E_{measured}$$

where *r* and *R* are the emitter-detector distance and the detector cross-section radius respectively. We compare this measurement with the signal collected by the fiber and driven to detector D2 on the oscilloscope. Both calibrations show very good linear fittings. Finally, the delay time between detectors D1 and D2 is also easily calibrated substituting the pump filter by another one for the emission.

**Figure 3:**
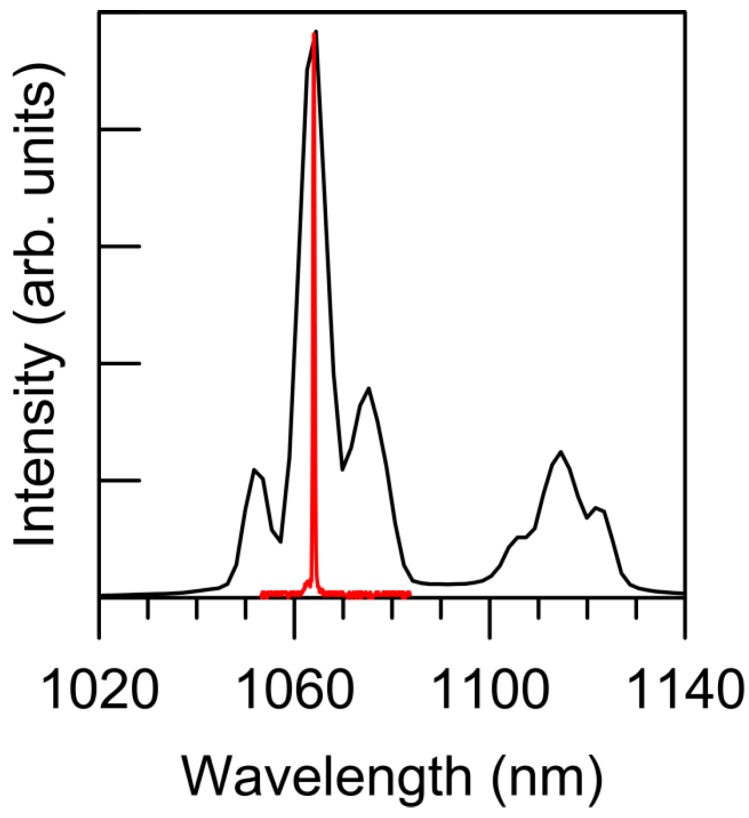
Spontaneous (**black line**) and stimulated (**red line**) emission spectra of Nd:YAG crystal powder (stimulated emission intensity is several orders of magnitude higher).

A typical threshold behavior has been obtained when pumping below or above a given pump energy. The emission spectrum narrows to a single peak down to the resolution of our measurement system (0.3 nm), as can be seen in Figure 3. The peak wavelength at 1064.1 nm does not change with the pump wavelength. The emission pulse duration shortens suddenly from about two hundred microseconds (spontaneous emission decay time) to a few nanoseconds (in the order of pump pulse duration), whereas the intensity of the emitted pulse increases by some orders of magnitude.

## 4. Results

**Figure 4:**
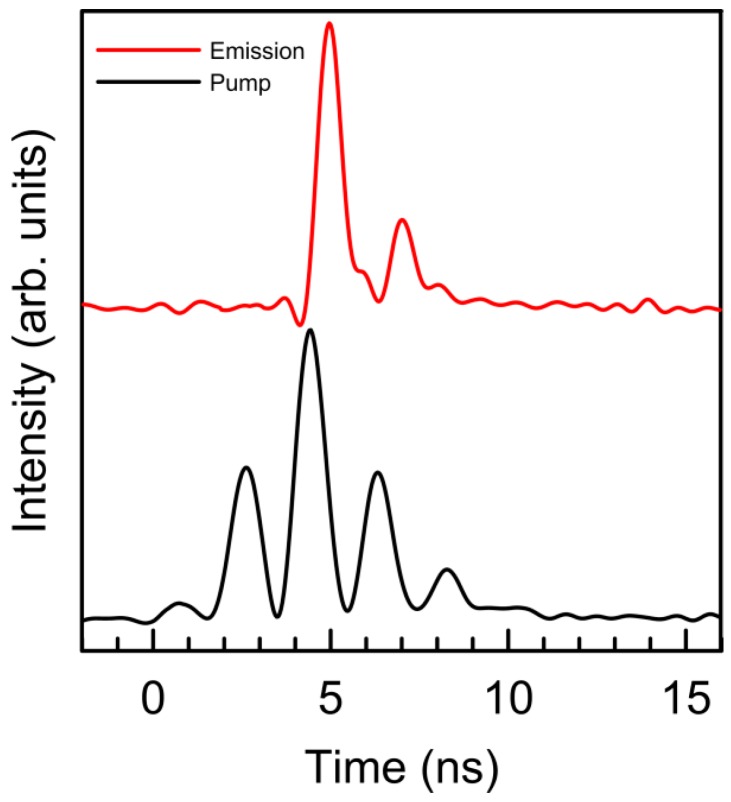
Pump pulse intensity (**black line**) and the corresponding output laser intensity (**red line**), as a function of time.

As an example, Figure 4 shows a pump pulse and the corresponding stimulated emission pulse as a function of time. As can be seen, at the forward side of the pump pulse, no stimulated radiation is emitted, because the pump energy is being absorbed to reach the threshold level of the population inversion (pulse buildup time). After that, the RL intensity follows the pump intensity fluctuations with a delay of about 500 picoseconds.

We have observed that, in Nd stoichiometric crystal powders with a similar grain size, this delay time is much shorter [34,35]. In regular laser systems, this delay time between pump intensity and emission fluctuations is determined by the residence time of the photon inside the resonator [36]. The residence time in RLs must be an intrinsic feature of multiple light scattering because, at last, the “resonator” is constituted only by scattering and absorption. In the low-doped Nd:YAG crystal powders, this response time and therefore the residence time of the photon inside the “multiple scattering resonator” is of the order of a few hundreds of picoseconds, much longer than in stoichiometric samples, due to its much lower absorption. In other words, the emitting volume and thus the path length of the photon in the “stochastic cavity” is demonstrably longer in low concentrated samples than in stoichiometric ones.

**Figure 5:**
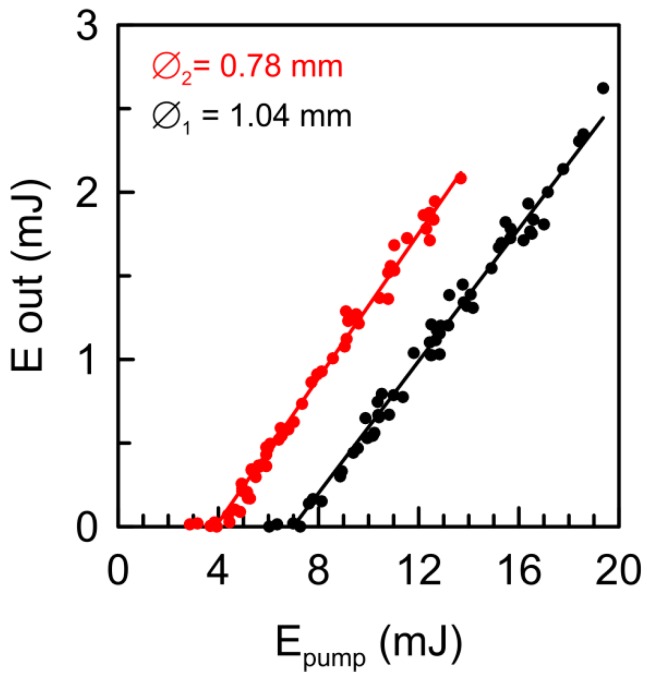
Input/output energy slope lines for Nd:YAG crystal powder pumped at 808.5 nm, for two different diameters of the pump beam: Ø_1_ = 1.04 mm (**black points**) and Ø_2_ = 0.78 mm (**red points**). Slope efficiency is about 20%, and thresholds are 7.1 and 4.0 mJ respectively.

The RL input/output energy slope curves are shown in Figure 5 for two different diameters of the pump beam: Ø_1_ = 1.04 mm and Ø_2_ = 0.78 mm.

As can be seen, the two slope efficiencies are equal for both pump beam sizes (20% ± 1%), although the pump beam areas are quite different. The value of the slope efficiency (*m*) agrees with the one predicted by the expression:

(2) $$m=\eta \frac{\nu_{em}}{\nu_{abs}}\;$$

given by the authors in previous works [32,33], that is, the absorbance of the sample (*η* = 0.27) times the ratio between the energies of absorbed and emitted photons.

The slope efficiency in the Nd:YAG crystal powder is lower than those of Nd in stoichiometric crystal powders with a similar grain size, which can achieve a value close to a 40% due to their higher absorbance peaks [33,35]. It is important to remark that this value of the slope efficiency means that every absorbed photon above the threshold energy is reemitted again as a stimulated emission photon. This is connected with Figure 4 in the sense that the stimulated emission time constant must be faster than all other loss channel rates.

On the other hand, the experimental pump threshold energies shown in Figure 5 (4.0 and 7.1 mJ) divided by their respective pump areas (0.48 mm^2^ and 0.85 mm^2^) approximately yield a constant value of 8.3 mJ/mm^2^. The constancy of the pump threshold energy density (pump energy by unit area) seems to be valid as far as a focusing limit is avoided. This result also agrees with some of our previous results [32,33] but is not so clear in other references in the literature, as, for example, in [37,38].

**Figure 6:**
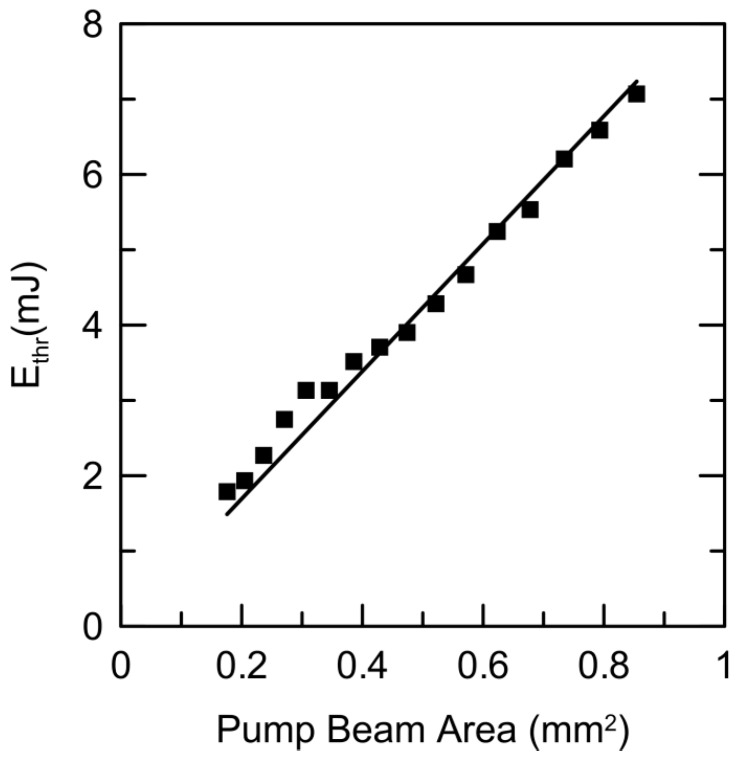
Threshold pump energy for Nd:YAG crystal powder as a function of pump beam area. As can be seen, the threshold energy fits well a linear function; therefore, the threshold energy density is constant (around 8.4 mJ/mm^2^). Slight deviations are observed at the smallest areas.

In order to clarify this issue, and to investigate the range of validity of the constancy of pump threshold energy density, we measured the threshold energy as a function of the pumped area for many different pump beam areas, by slowly moving the L focusing lens, starting with a pump spot with a diameter of 1.04 mm, down to 0.47 mm. The obtained result is displayed in Figure 6. It is worth noticing that, even at the widest pump beam size, the laser threshold energy can be reached at around 7 mJ, whereas the minimal threshold energy is achieved below 2 mJ with the most focused spot size. As can be seen, the threshold energy fits a good linear function when plotted *versus* the pump beam area, with a slope of 8.4 mJ/mm^2^, very close to the value deduced in Figure 5. Nevertheless, we found that, at the smallest pump areas (less than 0.4 mm^2^), the value of the threshold density presents some deviations, probably because the powder surface was somehow “marked” by the pump beam.

It is difficult to estimate the damage threshold due to an excessive pump energy density on the sample, because it is difficult to discriminate whether we are producing some kind of “burning” or “drilling” on it. It could be a combination of both. In any case, it limits the linear behavior of the RL threshold energy as a function of the pump beam area. The highest pump energy density (in a linear regime), shown in Figure 5, corresponds to around 30 mJ/mm^2^ with a 0.78-mm-diameter pump spot, and no marks were observed in the powder surface.

**Figure 7:**
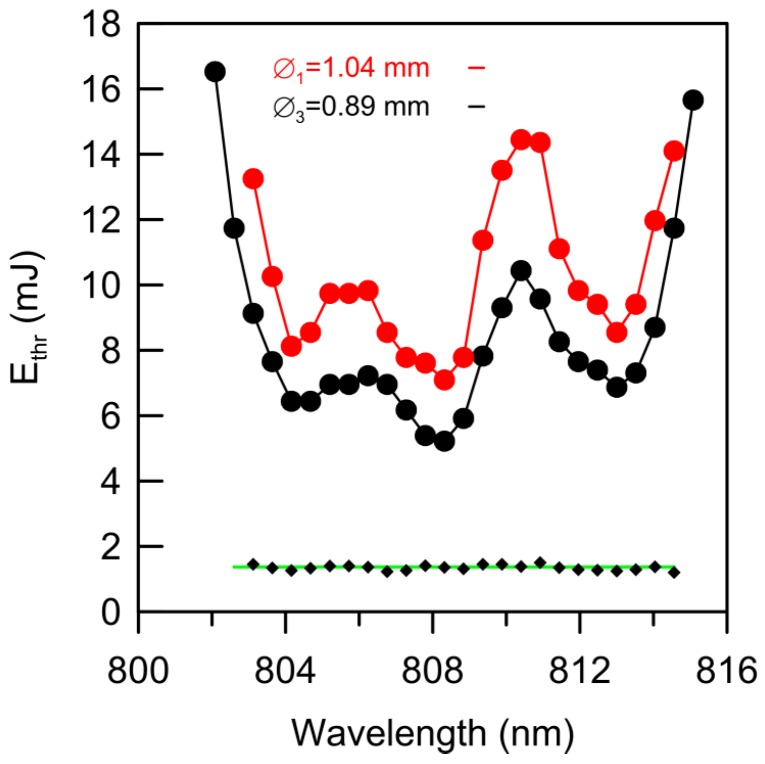
Threshold pump energy as a function of pump wavelength at two different pump beam diameters: Ø_1_ = 1.04 mm (**red**) and Ø_3_ = 0.89 mm (**black**). As can be seen, the ratio between both curves is nearly constant (**black diamonds**) and equal to the beam area ratio (**green line**).

Finally, Figure 7 shows the threshold pump energy as a function of the pump wavelength at two different pump beam diameters: Ø_1_ = 1.04 mm and Ø_3_ = 0.89 mm. As can be seen, the shape of both curves follows the absorbance variations shown in Figure 1. The bottom of Figure 7 shows the fitting of the calculated ratio between both curves (black diamonds), which is nearly constant and close to the ratio between both pump beam areas (1.37, green line). This result clearly demonstrates again that the pump threshold energy density is a constant, provided that the pump beam is not excessively focused.

## 5. Conclusions

We demonstrated here a room-temperature RL emission from a 1 mol % Nd-doped YAG crystal powder, without the aid of any internal mirror and with a threshold energy below 2 mJ. Second, we observed that the RL residence time (an intrinsic feature of the multiple light scattering) is, in this low-doped crystal, of the order of hundreds of picoseconds, longer than in stoichiometric crystals with a similar grain size. Third, we were able to measure the total emitted random stimulated emission energy and the absolute laser slope efficiency, which is about 20%. Fourth, we confirmed that the slope efficiency does not change with the pump beam size (provided that the pump energy density does not exceed a limiting value), because it only depends on sample absorbance and the ratio between absorbed and emitted photon energies. This conclusion means that every photon absorbed by the powder above the threshold value is reemitted again as a stimulated emission photon. Finally, we confirmed that the pump threshold energy is proportional to the beam area, insofar as the aforementioned limiting pump energy density is not surpassed.
